# Role of durotomy on function outcome, tissue sparing, inflammation, and tissue stiffness after spinal cord injury in rats

**DOI:** 10.1002/mco2.530

**Published:** 2024-04-04

**Authors:** Chen Jin, Kaiwei Wang, Yilong Ren, Yi Li, Zhanwei Wang, Liming Cheng, Ning Xie

**Affiliations:** ^1^ Key Laboratory of Spine and Spinal Cord Injury Repair and Regeneration of Ministry of Education Orthopaedic Department of Tongji Hospital School of Medicine Tongji University Shanghai China; ^2^ Department of Orthopedics Tongren Hospital Shanghai Jiaotong University School of Medicine Shanghai China; ^3^ Department of Orthopedics Shanghai General Hospital Shanghai Jiaotong University School of Medicine Shanghai China

**Keywords:** decompression, durotomy, functional recovery, secondary injuries, Spinal cord injury

## Abstract

Currently, there is a lack of effective treatments for spinal cord injury (SCI), a debilitating medical condition associated with enduring paralysis and irreversible neuronal damage. Extradural decompression of osseous as well as soft tissue components has historically been the principal objective of surgical procedures. Nevertheless, this particular surgical procedure fails to tackle the intradural compressive alterations that contribute to secondary SCI. Here, we propose an early intrathecal decompression strategy and evaluate its role on function outcome, tissue sparing, inflammation, and tissue stiffness after SCI. Durotomy surgery significantly promoted recovery of hindlimb locomotor function in an open‐field test. Radiological analysis suggested that lesion size and tissue edema were significantly reduced in animals that received durotomy. Relative to the group with laminectomy alone, the animals treated with a durotomy had decreased cavitation, scar formation, and inflammatory responses at 4 weeks after SCI. An examination of the mechanical properties revealed that durotomy facilitated an expeditious restoration of the injured tissue's elastic rigidity. In general, early decompressive durotomy could serve as a significant strategy to mitigate the impairments caused by secondary injury and establish a more conducive microenvironment for prospective cellular or biomaterial transplantation.

## Graphical abstract

Spinal cord injury (SCI) can cause permanent paralysis and lacks effective treatments currently. Here, we performed durotomy to (1) reduce tissue oedema; (2) decrease scar formation, cavitation, and inflammatory responses; (3) promote rapid recovery of stiffness of injured tissue. Our study provides compelling evidence elucidating the role of durotomy, which may also shed new light on the development of therapeutic strategy for SCI repair.

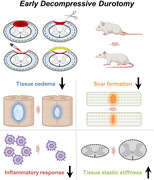

## 1 INTRODUCTION

Spinal cord injury (SCI), one of the most incapacitating neurological disorders that can result in limited movement, pain, and dysfunction of the autonomic nervous system, is a significant global issue in terms of public health. The worldwide incidence of SCI is estimated to be between 250,000 and 500,000 persons annually.^1^, ^2^, ^3^ Despite numerous fundamental scientific and clinical breakthroughs in the treatment of SCI, currently, there is a deficiency of a solitary efficient therapeutic strategy to avert the severe paralysis linked with this type of injury.

SCI frequently occurs in two stages. The initial stage involves the immediate obliteration of cells and tissues due to a single traumatic incident, including a car crash or sports‐related accident. The subsequent stage, known as the secondary phase, involves various cellular and molecular consequences of the initial injury, such as swelling, bleeding, inflammation, degeneration of nerve fibers, formation of scar tissue, and the development of cysts. These secondary effects lead to the enlargement of the initial injury site over a longer period of time, ranging from days to weeks.^4^, ^5^, ^6^ The injured spinal cord may become swollen inside the thecal sac due to the edema and hemorrhage, contributed to occlusion of the subarachnoid.^7^ Intraspinal pressure (ISP) is increased when the rigid dural sleeve is compressed against the enlarged, injured cord.^8^ The condition, which has been observed in pigs,^9^ humans,^10^ and rats^11^ is known as “spinal compartment syndrome.”^12^ Elevated ISP leads to a decline in self‐regulation of blood vessels and ineffective uneven blood flow in the injured spinal cord, worsening the impact of oxidative stress and subsequent harm.^9^ Recently, heightened ISP has been linked to adverse neurological consequences in individuals with SCI.^13^, ^14^, ^15^, ^16^ Moreover, the meticulous handling of escalated ISP following SCI seems to align with enhanced functional outcomes.^17^, ^18^

Through a deeper comprehension of the underlying mechanisms of acute SCI, it is believed that surgical decompression may help mitigate the impact of subsequent injuries.^19^ The primary approach in current surgical procedures typically includes relieving pressure on the bone canal through laminectomies, correcting the deformity, and stabilizing the spine.^20^ Osseous decompression alleviates pressure from edema and hemorrhage and prevents blood or oxygen shortages. However, this particular surgical procedure does not relieve elevated ISP, which is witnessed in roughly 50% of individuals with SCI.^10^ Therefore, to efficiently reduce immediately heightened ISP after SCI, it is imperative to perform a durotomy by opening the dural lining of the spinal cord. Moreover, based on a thorough examination of existing preclinical data, the timing of spinal cord compression emerges as a crucial factor influencing the recuperation process following SCI.^21^, ^22^ Following acute SCI, the first 24 h after injury are critical for achieving optimal neurological recovery with decompressive surgery.^22^, ^23^

Presently, there is ongoing debate regarding the prospective neurological benefits of durotomy. Smith et al.^24^ examined the recovery implications of durotomy with duroplasty in 72 rodents suffering from cervical SCI. The rats that underwent a dural incision and got a dural graft exhibited less cavitation, inflammation, and scar formation, as well as superior functional recovery in comparison with the group that only received contusion. Similarly, Zhang et al.^25^ conducted a recent study where they performed durotomy on rats. The researchers found that intrathecal decompression, achieved through a dural incision, led to enhanced neurological function. Microscopic investigations indicated that the rats exhibited more preservation of white matter, as well as a reduced presence of vacuoles and less damage to axons. Nevertheless, Jalan et al.^26^ suggested that surgical decompression did not yield any advantages in terms of neurological enhancement in rats with severe thoracic spinal cord contusion injury 24 h after the injury occurred. Overall, these findings only assessed the behavior test and/or conducted a single histological examination (without quantitative verification), but they did not offer enough information to elucidate the significance of early surgical decompression of the intradural region following SCI. Therefore, further investigation is recommended.

**FIGURE 1:**
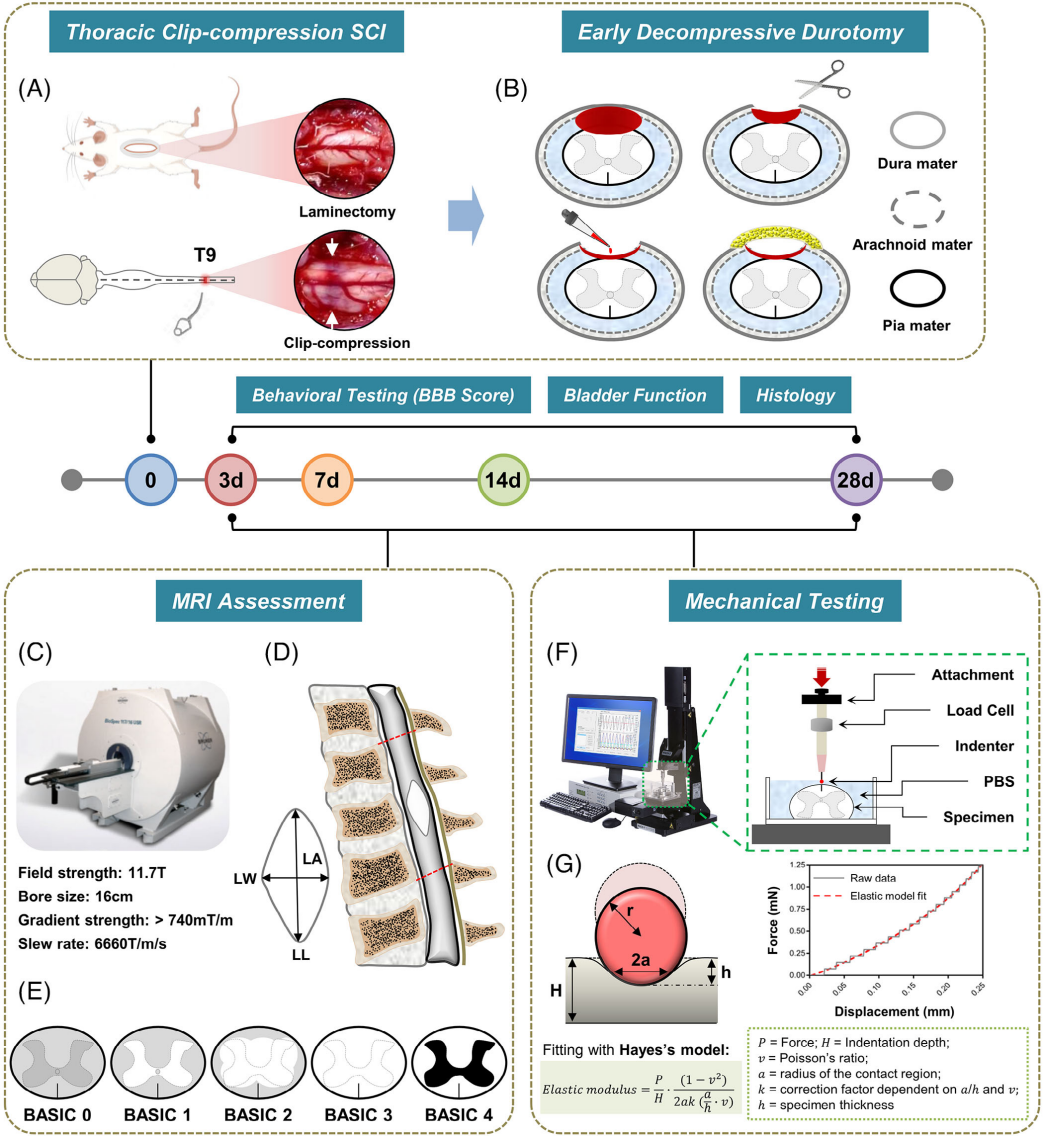
Schematic representation of experimental design. (A) Clip‐compression model for SCI. The process incorporates the following steps: dorsal incision, thoracic level T9 (ninth vertebra) laminectomy for spinal cord exposure, and extradural application of an aneurysm clip in the spinal cord. (B) Schematic representation of the decompressive durotomy technique. The first panel (top, left) = subdural edema and hemorrhage formation; the second panel (top, right) = durotomy; the third panel (bottom, left) = evacuation of hemorrhage and expansion of intradural space; the forth panel (bottom, right) = wound covered with a piece of adipose tissue. (C) Representation of small bore animal magnetic resonance imaging (MRI) scanner (Biospec 117/16; Bruker, Ettlingen, Germany). (D) Schematic representation of the MRI parameters of lesion region, which include lesion area, lesion length, and lesion width. Lesion area represents the proportion of the lesion area in the T8–T10 spinal cord, and lesion length and lesion width represent the maximum longitudinal and transverse diameters of the lesion area, respectively. (E) BASIC scoring system upon axial T2‐weighted MRIs. BASIC 0 = normal, BASIC 1 = T2 hyperintensity confined to gray matter, BASIC 2 = T2 hyperintensity involving gray matter with some white matter, BASIC 3 = T2 hyperintensity involving the entire transverse extent, BASIC 4 = grade 3 with hemorrhage. (F) Experimental setup for ex vivo spinal cord tissue indentation testing. Test apparatus shows indentation testing rig and assistive devices. The dashed rectangle depicts schematic of indentation testing setup. (G) Schematic representation of indentation process (the left panel). The force–displacement curves obtained by indentation tests followed a spherical Hayes's contact model (the right panel). The elastic modulus is obtained using the thickness and an elastic model in spherical indentation.

In the current study, we propose to comprehensively ascertain the potential implications of surgical decompression with early durotomy in the treatment of acute thoracic SCI and its impacts on functional recovery, edema, inflammation, scar formation, cystic cavitation, and tissue elastic stiffness based on a combination of behavioral analyses, radiological assessment, histopathological examination, and mechanical indentation testing (Figure 1). The study hypothesized that durotomy conducted 24 h postinjury would enhance functional, histological, radiological, and tissue mechanical property outcomes in an animal model of acute severe thoracic SCI.

## 2 RESULTS

### 2.1 Durotomy improved locomotor function and bladder recovery after SCI

**FIGURE 2:**
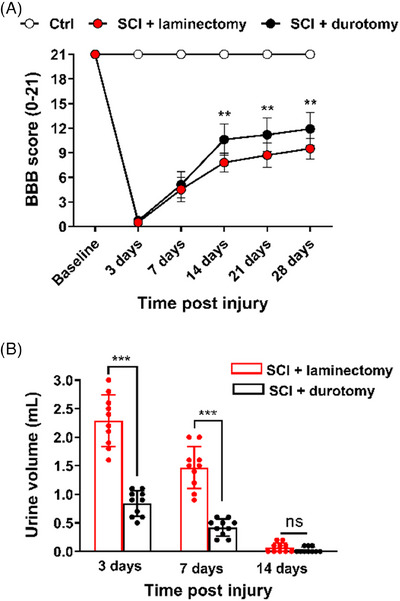
Behavior testing and bladder function assessment after SCI. (A) All injured animals showed spontaneous recovery of their hindlimb open‐field locomotion to a certain extent. SCI + durotomy leads to significantly better overall Basso–Beattie–Bresnahan (BBB) scores compared with SCI + laminectomy. No significant differences in BBB scores were found among the three groups in the preinjury period. Ctrl, the control group. (B) SCI + durotomy resulted in earlier bladder reflex recovery compared with SCI + laminectomy. Values are expressed as the mean ± standard deviation (*N* = 10 animals per group per time point). ^**^ indicates *p* < 0.01 and ^***^ indicates *p* < 0.001.

There were no notable variations in the preinjury Basso–Beattie–Bresnahan (BBB) scores between the groups, as depicted in Figure 2A. Throughout the duration of the study, there were no observed alterations in hindlimb locomotor function among the sham control group. The BBB scoring performed 7 days following SCI manifested that the animals that had received a durotomy had better but not statistically significant motor functional recovery than those that had received a laminectomy alone (5.1 ± 1.6 compared with 4.5 ± 1.5, *p* = 0.694). The variation between SCI + durotomy and SCI + laminectomy groups was significant at 14 days (10.6 ± 1.9 compared with 7.8 ± 1.1, *p* = 0.001), 21 days (11.2 ± 2.0 compared with 8.8 ± 1.4, *p* = 0.007), and 28 days (11.9 ± 2.0 compared with 9.5 ± 1.3, *p* = 0.005) (Figure 2A). None of the animals in either group fully recovered their baseline levels.

A common characteristic of thoracic crush models is that rats are unable to voluntarily empty the bladder following an injury. During the initial 14‐day period, we assessed the remaining urine quantities in all animals following laminectomy and durotomy procedures. The urine volume of the SCI + laminectomy group was significantly greater than the SCI + durotomy group at 3 days (2.29 ± 0.45 compared with 0.84 ± 0.22, *p* < 0.001) and 7 days (1.47 ± 0.37 compared with 0.42 ± 0.15, *p* < 0.001). No significant variation between SCI + laminectomy and SCI + durotomy groups at 14 days (0.07 ± 0.08 compared with 0.03 ± 0.05, *p* = 0.202). Interestingly, the results demonstrated that durotomy plus laminectomy led to significantly faster recovery of bladder function than laminectomy alone (*p* < 0.001) (Figure 2B).

### 2.2 Durotomy reduced lesion size and tissue edema after SCI

**FIGURE 3:**
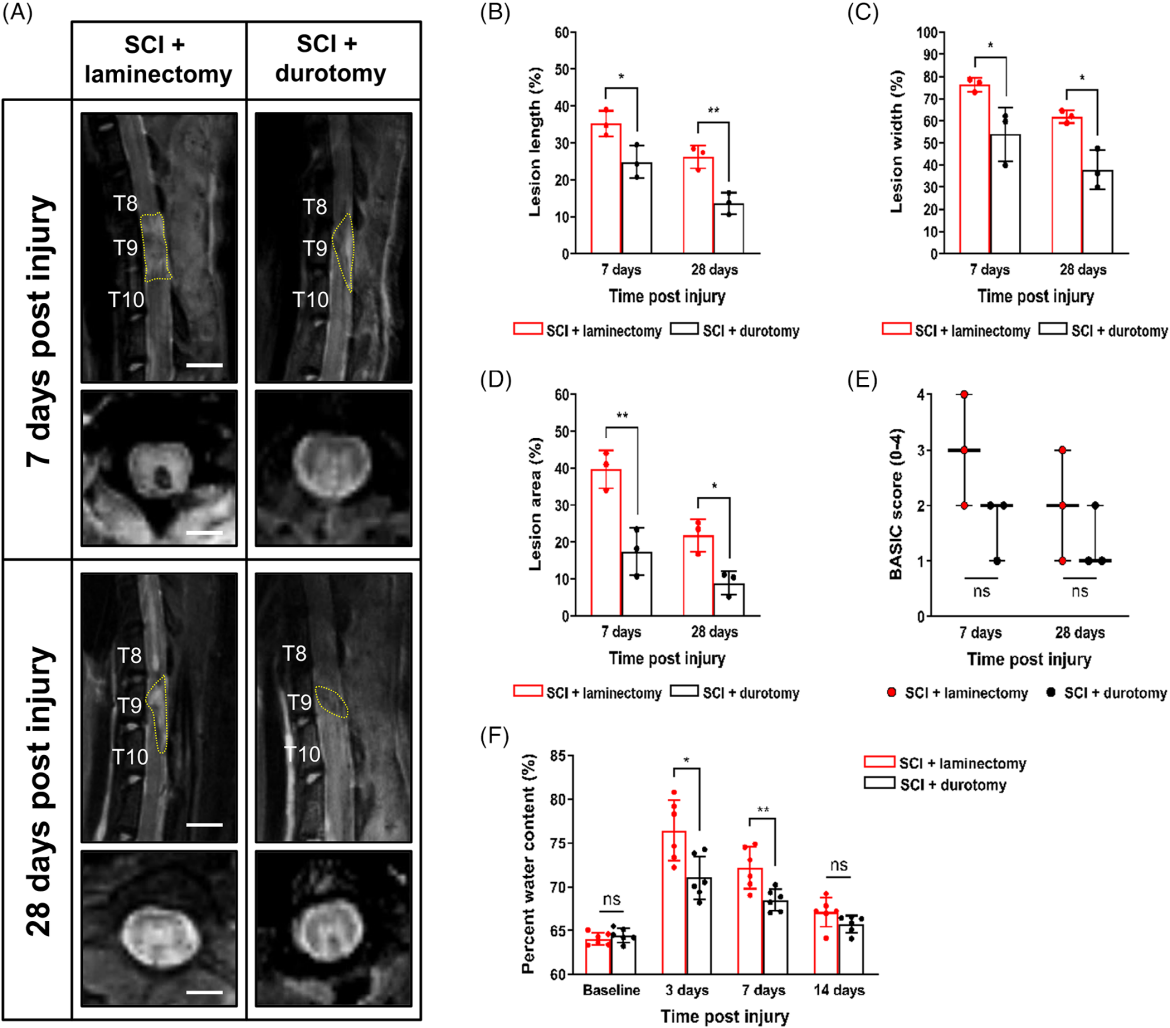
MRI parameters analysis and tissue percent water content assessment after SCI. (A) Sagittal and axial T2‐weighted images of rats in two groups on days 7 and 28 postinjury. For better delineation, the peripheral margins of the lesion site are outlined in yellow on sagittal T2‐weighted images. (B–D) Comparison between two groups on lesion length, lesion width, and lesion area on days 7 and 28 postinjury, respectively. Values are expressed as mean ± standard deviation (*N* = 3 animals per group per time point). ^*^ indicates *p* < 0.05 and ^**^ indicates *p* < 0.01. Exact *p* values for (B–D) are listed in Table S1. (E) Comparison between two groups according to BASIC scores on days 7 and 28 postinjury. In all graphs, the upper and lower lines indicate maximum and minimum values, and the middle line shows the median. *N* = 3 animals per group per time point. (F) Comparison between two groups on percent water content at 3 days, 7 days, and 14 days postinjury. Values are expressed as mean ± standard deviation (*N* = 6 animals per group per time point). ^*^ indicates *p* < 0.05 and ^**^ indicates *p* < 0.01. Scale bars = 10 mm in A. Exact *p* values for (F) are listed in Table S2.

Different lesion regions (i.e., edema [high signal intensity] and/or subdural hemorrhage [low signal intensity]) of the spinal cord in the SCI + laminectomy and SCI + durotomy groups were found in sagittal and axial T2‐weighted images (Figure 3A). Quantitative analysis revealed that lesion length, lesion width, and the metrices of the lesion area were significantly less in the group that had durotomy compared with the SCI + laminectomy group on days 7 and 28 postinjury (*p* < 0.05; Figures 3B–D; Table S1). Furthermore, four distinct patterns of intramedullary signals (i.e., Brain and Spinal Injury Center [BASIC] 1−4) were identified on the axial T2‐weighted sequence at the injury epicenter. No statistically significant difference was found in the BASIC score between the two groups on days 7 and 28 postinjury (*p* > 0.05; Figure 3E).

Edema is suggested by the presence of fluid accumulation in the white matter surrounding the lesion site throughout the acute phase after the initial injury, as indicated by the increased T2 signals. We reasoned that local tissue edema was alleviated by early intrathecal decompression. To test this, we compared the percent water content of the ∼20.0 mm cord segment centered over the lesion center between the SCI + laminectomy and SCI + durotomy groups and found that SCI gave rise to significantly pronounced tissue edema compared with baseline in both groups. On day 3, the water content of the lesion center in the SCI + laminectomy group had increased to an average of 76.4 ± 3.5%, which was notably higher than that in the SCI + durotomy group (71.0 ± 2.5%, *p* = 0.011; Figure 3F; Table S2). By day 7, tissue edema at the lesion center in rats that had received a crush SCI alone was still significantly higher than that in rats that had received an early decompression (72.2 ± 2.4% compared with 68.5 ± 1.3%, *p* = 0.007; Figure 3F; Table S2).

### 2.3 The impact of durotomy on reactive astrogliosis, macrophage accumulation, as well as extracellular matrix deposition following SCI

**FIGURE 4:**
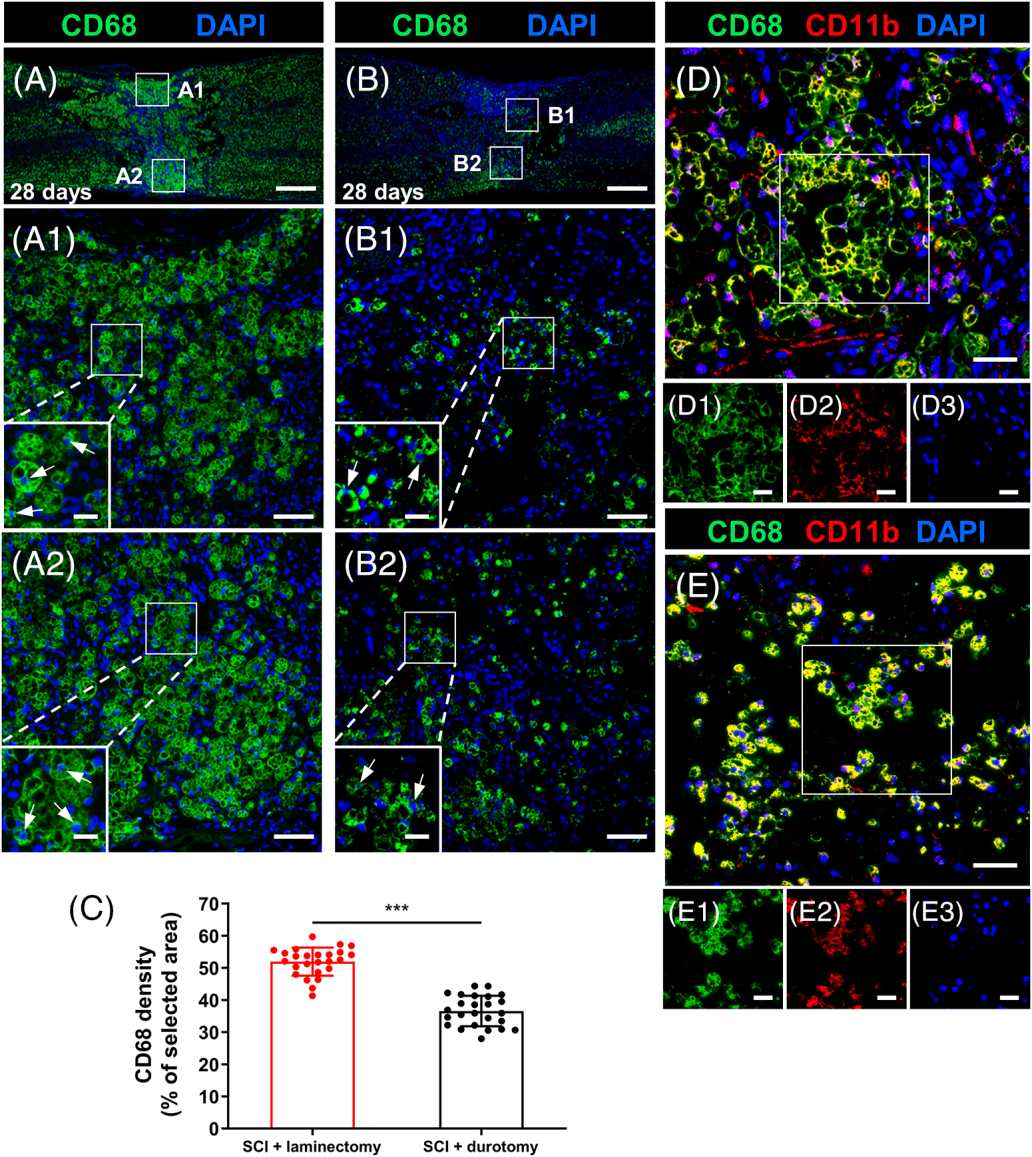
Effects of durotomy on macrophage accumulation after SCI. (A and B) CD68 immunostaining images of longitudinal sections of spinal cord segments from the SCI + laminectomy group and SCI + durotomy group. At 28 days postinjury, the animals that had received a decompressive durotomy showed a decreased inflammatory response relative to that in the SCI + laminectomy group. (A1), (A2) and (B1), (B2) showing high‐magnification images of the boxes in (A) and (B), respectively; arrows indicate CD68‐positive cells (macrophages). (C) Bar chart showing the stereoscopic quantification results of CD68 immunofluorescence density (percentage of CD68‐immunopositive area per selected area) in the SCI + laminectomy group and SCI + durotomy group. Values are expressed as mean ± standard deviation (*N* = 5 animals per group). (D and E) Double immunostaining for CD68 (green) and CD11b (red) at the injury site from the SCI + laminectomy group and SCI + durotomy group. DAPI, a nuclear dye, is used for counterstaining (blue). ^***^ indicates *p* < 0.001. Scale bars = 500 µm in (A and B), 50 µm in (A1, A2, B1, B2, C, and D), and 20 µm in boxes of (A1, A2, B1, B2, C1, C2, C3, D1, D2, and D3).

After a period of 28 days following SCI without any additional treatments, there was a significant presence of CD68‐positive macrophages observed through stereological quantification in the area surrounding the lesion site (Figure 4). Furthermore, a great number of macrophages were observed near and far from the injury site along the white matter pathways undergoing anterograde (Wallerian) degeneration (Figures 4A and B). Figure 4C shows that there were considerably fewer CD68‐positive macrophages in the SCI + durotomy group contrasted with the SCI + laminectomy group, both within and around the lesion (*p* < 0.001). The presence of microglial activation was further confirmed by double staining using immunostaining for CD68 and CD11b, two widely recognized markers of active microglia. The results showed that most of the CD68‐positive cells in the epicenter and surrounding the cystic cavities simultaneously expressed CD11b, indicating that they were activated microglial cells (Figures 4D and E).

**FIGURE 5:**
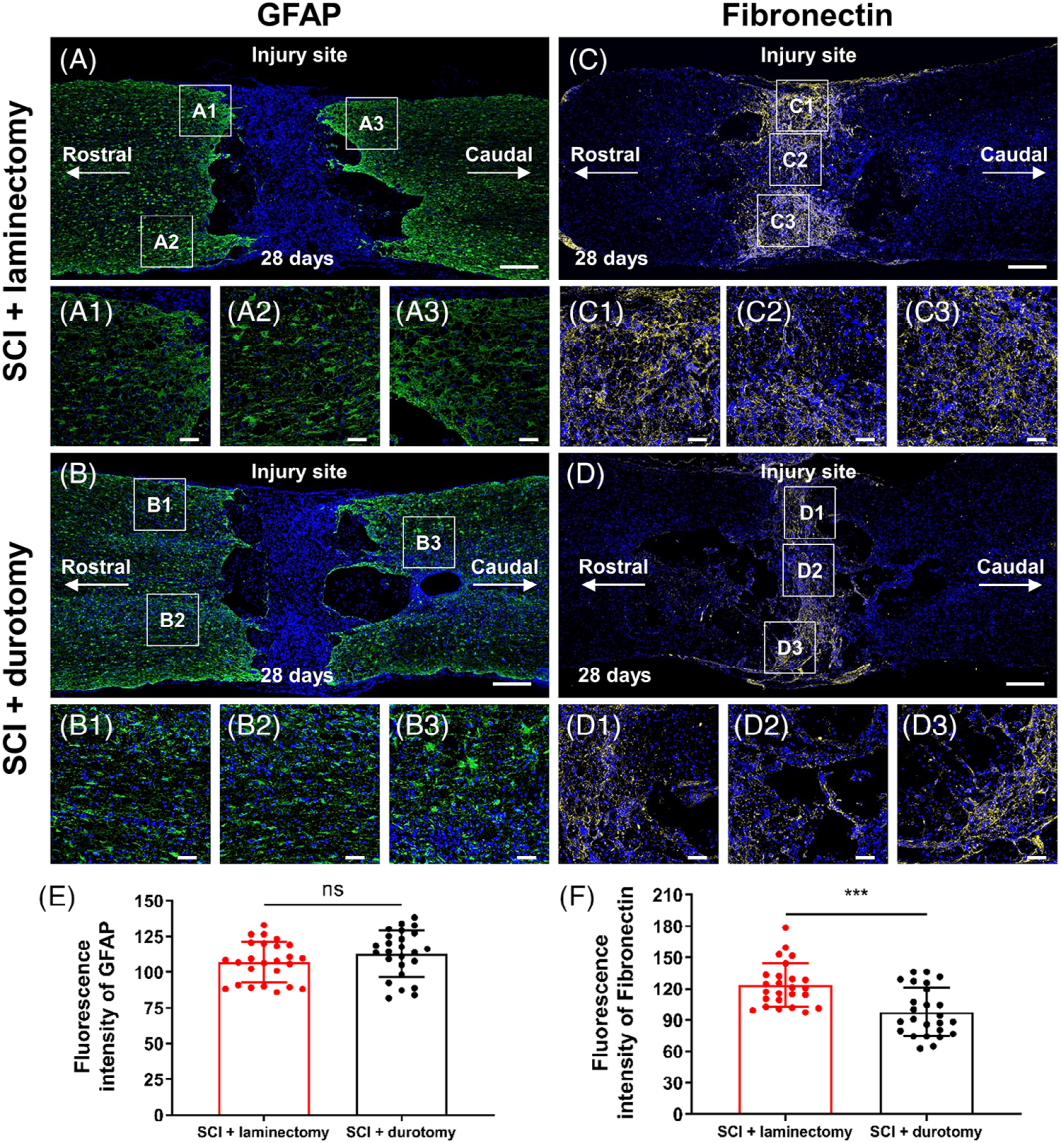
Effects of durotomy on fibroglial scar formation after SCI. (A and B) Immunohistochemical analysis with antiglial fibrillary acidic protein (GFAP) in the SCI + laminectomy and SCI + durotomy group. At 28 days postinjury, the extensive astrocyte proliferations are displayed in both SCI + laminectomy group and SCI + durotomy group. (A1–A3) and (B1–B3) show higher‐magnification images corresponding to the boxed areas in (A) and (B), respectively. (C and D) Immunohistochemical analysis with antifibronectin in the SCI + laminectomy group and SCI + durotomy group. At 28 days postinjury, the SCI‐only animals display the more extensive fibronectin expression, indicating greater fibrous scarring relative to that in the early decompressive durotomy group. (C1–C3) and (D1–D3) showing higher‐magnification images corresponding to the boxed areas in (C) and (D), respectively. (E and F) Bar chart showing the stereoscopic quantification results of GFAP and fibronectin immunofluorescence density in the SCI + laminectomy and SCI + durotomy groups, respectively. Values are expressed as mean ± standard deviation (*N* = 5 animals per group). ^***^ indicates *p* < 0.001. Scale bars = 500 µm in (A, B, C, and D) and 50 µm in (A1–A3, B1–B3, C1–C3, and D1–D3).

The study also examined the effect of durotomy on the degree of reactive astrogliosis, which was quantified by staining glial fibrillary acidic protein. On the 28th day after the injury, a significant number of hypertrophic astrocytes expressing GFAP protein were observed around the lesion in both experimental groups (Figures 5A and B). The control lesions in the SCI + laminectomy group were surrounded by reactive astrocytes, as indicated by the strong expression of GFAP around the cystic cavities (Figure 5A). While the occurrence of cystic cavitation was reduced in the spinal cords treated with durotomy, the presence of perilesional GFAP was still detected. The quantitative analysis revealed no significant disparity in the quantity of GFAP‐positive astrocytes between the two groups. This suggests that durotomy may not have an impact on reactive astrogliosis following SCI (*p* = 0.192; Figure 5E).

Fibronectin was used to characterize the spatiotemporal distribution of extracellular matrix and fibrous scars in the injured region of the spinal cord after durotomy. Twenty‐eight days postinjury, there was a notable presence of fibronectin in all spinal cord lesions, as well as in a widespread connective tissue matrix in both the SCI + durotomy and SCI + laminectomy groups. In the SCI + laminectomy group, a fibronectin connective tissue band was discovered, which connected the dural leaflets and spanned across the two sides of the initial crush injury (Figure 5C). In the SCI + durotomy group, marked fibronectin‐positive areas became more concentrated toward the central core lesion because of the dural defect (Figure 5D). Moreover, fibronectin expression in the SCI + laminectomy group was much heightened compared with the durotomy treatment group (*p* < 0.001; Figure 5F), indicating less dense fibrous scar formation after early intrathecal decompression.

### 2.4 Durotomy reduces cavity formation after SCI

**FIGURE 6:**
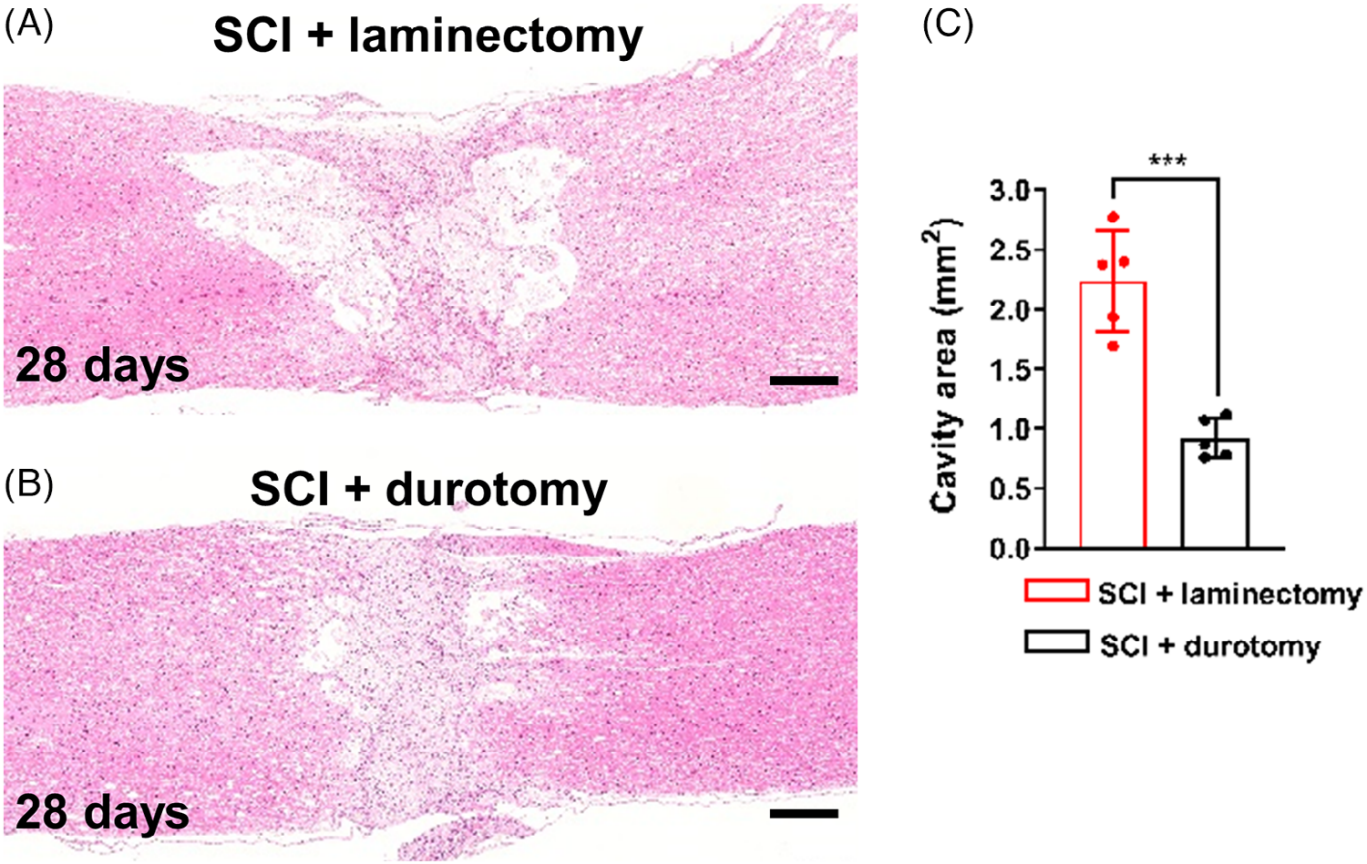
Reduction of cavity formation after SCI by durotomy treatment. (A and B) Hematoxylin and eosin staining images showing the cavities in the injured area of the spinal cord in the SCI + laminectomy and SCI + durotomy groups on day 28 postinjury. Scale bars = 500 µm. (C) Bar chart showing the cavity areas in the two groups. The cavity area measurements demonstrated significantly increased cavitation size at day 28 in the SCI + laminectomy group, which was relative to the size in the animals that had received an early decompressive durotomy. Values are expressed as mean ± standard deviation (*N* = 5 animals per group). ^***^ indicates *p* < 0.001.

Stereological analysis was conducted on crush injuries, both with and without durotomy, to assess the degree of cystic cavitation and investigate the potential impact of early intrathecal decompression on the formation of cystic cavities. After 28 days of SCI, a histological examination showed a concentrated cellular infiltration in the spinal cord lesion. Throughout the area represented by the dorsal columns and dorsal spinal gray matter, Figure 6A revealed extensive cystic cavities with many septae in the tissues of the spinal cords of patients with SCI. In contrast, durotomy led to a noticeable reduction in cystic cavitation, with only a few small microcystic formations remaining at the site of the lesion (Figure 6B). Quantitative comparative analysis of the LAs revealed that the spinal cord cavity area in the SCI + durotomy group was significantly smaller than that in the SCI + laminectomy group (*p* < 0.001; *N* = 5 per group; Figure 6C), suggesting that early intrathecal decompression may reduce cavity formation after SCI.

### 2.5 Effect of durotomy on tissue elastic properties of the spinal cord after SCI

**FIGURE 7:**
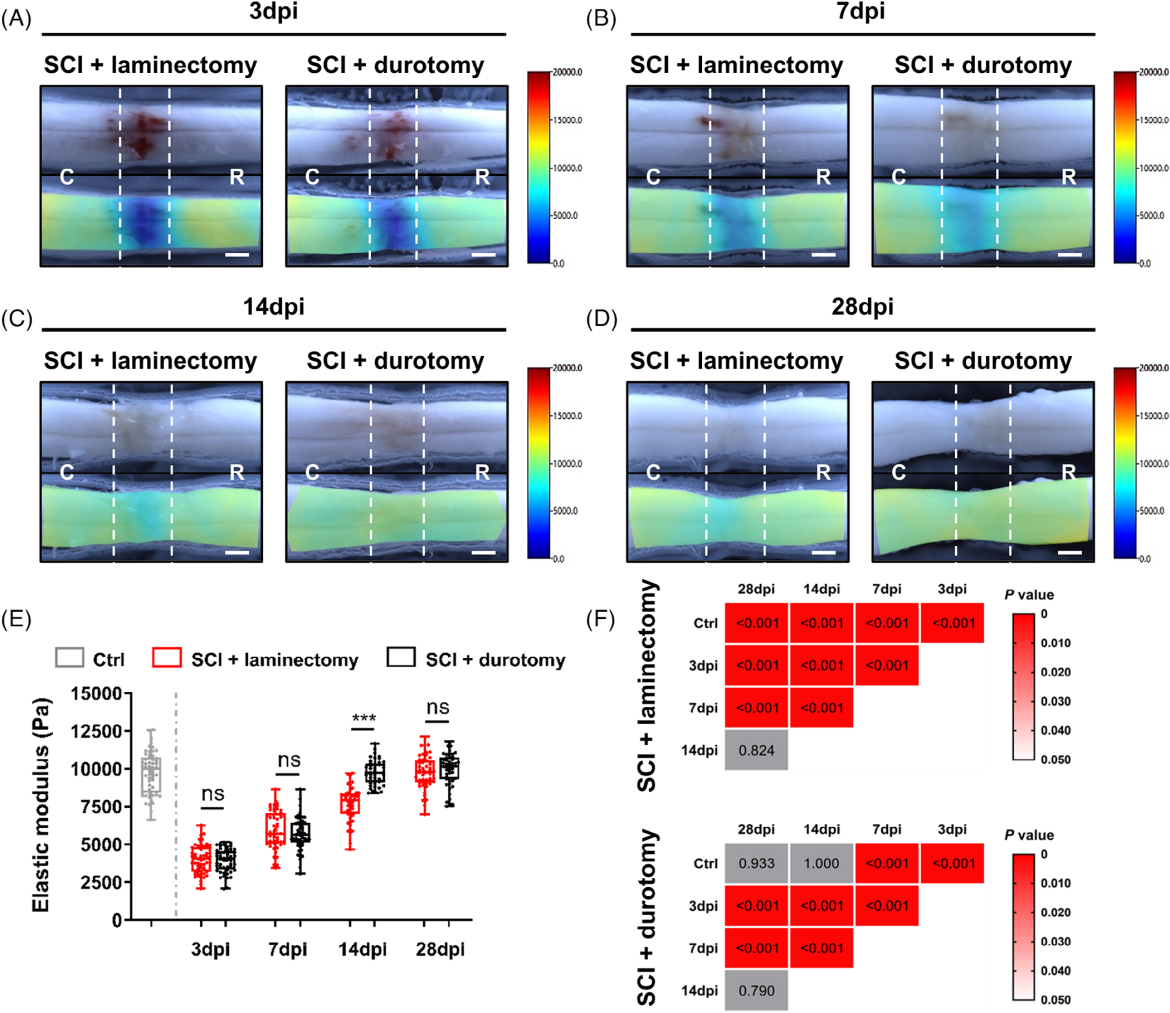
Effects of durotomy on tissue elastic properties of spinal cord after SCI. (A–D) The elastic moduli changes of spinal cord tissue at each time point postinjury. The color map of each group at each time point postinjury represents the spatial distribution of elastic stiffness. The white dotted line indicates demarcation between the injury region and away from injury region. The scale bar has a gradient of color from blue for the lowest elastic values (0 Pa) to red for the highest values (20,000 Pa) and yellow for the intermediate values (approximately 10,000 Pa). C = caudal, R = rostral directions. Scare bars = 1000 µm. (E) Comparison of the elastic moduli at each time point after injury between SCI + laminectomy and SCI + durotomy group. In all box plots, the top and bottom of the box represent the 75th and 25th percentiles, respectively, and the line inside the box corresponds to the median. Ctrl, the control group. Dpi, days postinjury. ^***^ indicates *p* < 0.001. Exact *p* values for (E) are listed in Table S5. (F) Statistical comparison by Kruskal–Wallis ANOVA of the elastic moduli of injured cord tissue among groups at different time points after injury, followed by Games–Howell's post hoc tests. The exact *p* value is showed in the individual square. Red and gray blocks indicate *p* < 0.05 and *p* > 0.05, respectively. Ctrl, the control group; Dpi, days postinjury.

The tissues were categorized into two segments based on their visual characteristics: the injury region (IR), which had a width of roughly 2.0 mm and encircled the central part of the lesion, and the area outside the injury region (AFIR). Throughout all the time points analyzed following the injury, there was no discernible disparity in mechanical properties between the healthy tissue of sham‐controlled rats and the uninjured spinal cord tissue in the AFIR of rats that underwent SCI alone or durotomy (*p* > 0.05; Figure S1). Indentation stiffness maps of the IR on day 3 following injury demonstrated a significant decline in tissue elasticity in the SCI + laminectomy and SCI + durotomy groups, with a decline of over twofold the amount of the uninjured region or control animals (*p* < 0.001; Figures 7A and S2; Tables S3 and S4). The morphology of the injured lesion with a diffuse margin was shown in the elastic modulus mapping. Following that, the spinal cord tissue in the injured region of both groups exhibited a gradual rise in flexibility in comparison with the unaffected area on the seventh day after the injury (*p* < 0.001; Figures 7B and S2; Tables S3 and S4). On the 14th day, there was still a notable disparity in the elastic modulus between the injured tissue in animals that only received SCI (*#*< 0.001; Figures 7C and S2; Tables S3 and S4). Animals that underwent durotomy showed similar results in the ex vivo specimens and stiffness maps. Although there were visible faint red marks and beginning scar development in the lesion core and rim areas of the damaged tissue, the stiffness maps did not indicate a significant disparity (*p* = 0.467; Figures 7C and S2; Table S4). On day 28, the measured elastic moduli of IR in the SCI + laminectomy group had increased, but tissue in AFIR remained stiffer than injured tissue (*p* < 0.001; Figure 7D; Figure S2; Table S3). However, we observed no significant difference in tissue elastic stiffness between the IR and AFIR tissues in the SCI + durotomy group (*p* = 0.734; Figures 7D and S2; Table S4).

As illustrated in Figure 7E, the elasticity evolution of the IR tissue displayed a nonlinear increasing trend in the two groups from day 3 to day 28 postinjury. Within the first week after SCI, days 3 and 7 were selected to observe and compare the extent of tissue elastic property recovery between the two groups; the findings revealed no obvious differences between them at day 3 or 7 postinjury (3986.9 ± 938.9 vs. 3943.4 ± 775.9 Pa, *p* = 0.796; 5951.47 ± 1253.6 vs. 5706.2 ± 1081.3 Pa, *p* = 0.293; Figures 7E and F; Table S5). Surprisingly, the spinal cord tissue recovered to basal levels more quickly over time in animals that had received durotomy treatment than in laminectomy‐only animals. The statistical analysis indicated that the elastic moduli of injured spinal cord tissue in SCI + durotomy group recovered toward 9733.5 ± 816.2 Pa at day 14 postinjury, with no notable difference compared with that of the control animals (*p* = 1.000; Figure 7F) but significantly higher compared with that of the SCI + laminectomy group (9733.5 ± 816.2 vs. 7657.4 ± 1141.3 Pa, *p* < 0.001; Figure 7E; Table S5). Little change was observed in tissue elasticity in the SCI + durotomy group for the following 14 days. No significant disparity between the sham‐controlled animals and durotomy‐treated animals was found (*p* = 0.933; Figure 7F). However, the tissue stiffness in the SCI + durotomy group remained stiffer than that of the laminectomy‐only animals at day 28 post‐SCI (9955.0 ± 1087.2 vs. 7889.8 ± 1039.2 Pa, *p* < 0.001; Figures 7E and F; Table S5).

## 3 DISCUSSION

The current research examined the impacts of an initial incision in the dura mater, which was done by creating a stable rat SCI model through crushing. Our experimental data suggest that decompression with laminectomy, followed by durotomy, provides significant functional benefits. Furthermore, based on a comprehensive analysis of radiographic, histological, and mechanical data, we found that early surgical decompressing of the intradural space limited the development of local edema and cystic cavitation, attenuated secondary inflammatory infiltration, and fibrous scar formation and promoted rapid recovery of the elastic properties of injured tissue.

Secondary SCI in animals and humans is commonly attributed to edema.^27^, ^28^, ^29^, ^30^ Vasogenic edema is characterized by the disruption of the blood‐brain barrier, resulting in the escape of plasma fluid into the extracellular space.^31^ Following SCI, the enlarged spinal cord might exert pressure on the dural components, leading to elevated cord pressure within the tissue (due to the thicker and stiffer pial‐glial zone in the spinal cord contrasted with the brain, the cord is more susceptible to pressure effects).^30^ The animals that received early decompressive durotomy exhibited significant parenchymal edema, as the spinal cord partly ruptured via the durotomy site during decompression, which was visible to the naked eye. Further, the findings of percentage water content showed that SCI resulted in notable tissue swelling, which was particularly evident compared with the initial state for both groups during the initial 2‐week period following the injury. Despite the absence of a notable distinction on day 14, the initial phases of SCI resulted in detrimental impacts on the recuperation of behavior due to the severity of tissue damage and compression of interstitial edema. These effects can be detected through BBB evaluation and were more pronounced compared with the SCI + durotomy group. The results demonstrated that early intrathecal decompression may play a more significant role in preventing secondary injury caused by compression of a swollen spinal cord.

Clip‐compression damage to the spinal cord may lead to the infiltration of both acute and chronic inflammatory cells. The initiation of posttraumatic cystic cavitation has been demonstrated to occur when peripheral macrophages and resident microglia infiltrate and become activated after SCI.^32^ In the present investigation, the occurrence of cystic cavitation was almost non‐existent in instances where durotomy was employed, potentially due to a significant hindrance in the buildup of macrophages at the injury site. Extensive infiltration of macrophages occurred in the SCI + laminectomy group, potentially leading to the development of sizable cystic cavities at the site of injury.

As far as we know, there has been no investigation into the alteration of the elastic rigidity of damaged cord tissue following durotomy. During the initial 14 days after the injury, we noticed a significant decrease in stiffness at the epicenter in both groups. The stiffness reached its minimum on the 3rd day following the injury, potentially attributable to the development of tissue swelling at the location of the acute phase. This significant observation largely corroborated previous findings by Moeendarbary et al.,^33^ who also documented a substantial decrease in the rigidity of rat spinal cord tissue roughly 1.5 weeks after the traumatic lesion. Our results also indicated that 14 days after the injury, the stiffness of the damaged tissue in animals that underwent early durotomy eventually approached levels similar to those of the uninjured tissue, which were higher than those in animals that underwent SCI plus laminectomy. Remarkably, on day 28, the stiffness levels in the SCI + durotomy group exhibited no statistically significant difference compared with the SCI and control groups and remained similar to the baseline values. These findings are captivating due to several factors. The mechanical features of the microenvironment can affect the behavior and characteristics of nearby cells. Stiffness, for instance, modifies the process of cell differentiation,^34^ proliferation,^35^ and migration.^36^, ^37^, ^38^ The physical features of a substrate or tissue, such as its mechanical characteristics, can have an impact on the behavior of neurons. These qualities can affect many features, such as the rate at which neurites extend,^39^ the direction of axonal outgrowth,^40^ and the degree of branching in neurites.^41^ Furthermore, on a larger scale, any excessive forces that occur at the interface of tissues can disturb the overall structure and impede the movement of cells or axons. Furthermore, the impact of injury on the mechanical properties of central nervous system (CNS) tissue may have substantial implications for neuronal regeneration despite the fact that a complete characterization of these effects is still lacking.^42^ In the present study, the behavioral results showed a pronounced difference between the two groups from day 14 postinjury, suggesting that earlier recovery of injured tissue stiffness resulted in better recovery of motor function. The rats that had undergone dural decompression showed clear functional indications of neuroprotection.

Thus far, there have been inconsistent reports about the impact of durotomy or duroplasty on functional outcomes.^24^, ^25^, ^26^, ^43^ Animal investigations have demonstrated the beneficial impact of durotomy and duroplasty. The research investigation carried out by Smith et al.^24^ investigated the impact of performing a surgical procedure known as durotomy with duroplasty on the recuperation of rats with cervical SCI. The rats that underwent a dural incision and dural graft showed less inflammation, scar formation and cavitation compared with the group that just had a contusion. Additionally, they demonstrated enhanced functional recovery. Nevertheless, numerous research studies have documented adverse outcomes. According to Jalan et al.,^26^ they carried out durotomy and duroplasty procedures in rats but found no enhancement in mobility. The analysis of the rats' catwalk gait revealed a decrease in limb coordination. Furthermore, the dural transplant group exhibited greater lesions, heightened white matter, and additional compression following the placement of the graft. Camlar et al.^43^ analyzed the effects of durotomy on neurological function. The groups did not show any significant differences in Tarlov test scores, and the results of duroplasty were not positive. Even though expansile duroplasty is a commonly performed clinical procedure involving an alloderm graft that is sewn in, its standardization in experimental SCI literature has not been established. Although technically possible, expansile duroplasty with primary dural closure is generally not carried out in rats. This article utilizes a fat pad as an epidural graft, similar to the dural closure procedure used in pituitary surgery. Prior studies with rodents have utilized fibrin sealants, allografts, or collagen matrices. Complications in rodent experiments have been associated with many occurrences of dural closures. For example, Jalan et al.^26^ discovered that the utilization of readily accessible dural sealant can lead to substantial compression of the spinal cord in approximately 50% of the animals. Hence, it is inadvisable to utilize these sealants for the purpose of healing dural lesions in a rodent model of SCI. Despite numerous studies highlighting the beneficial impacts of duroplasty on functional outcomes, there remains a prevailing perception of its potential risks, leading certain surgeons to opt against conducting such procedures. It is worth mentioning that there is a lack of adequate clinical data, and it will be necessary to conduct a randomized controlled study in the future to ascertain the efficacy of durotomy.

This study has several important limitations. First, the small number of experimental animals is not enough to avoid selection bias. Second, prior to the production of crush SCI, the SCI protocol necessitated decompressive laminectomy; however, this does not accurately replicate a genuine clinical SCI scenario where a displaced spinal column could lead to initial injury and subsequent cord compression. Third, despite the extensive use of ex vivo measurement methods to gather most of the information regarding the mechanical properties of spinal cord tissue, this study has limitations due to the inability of ex vivo analyses to accurately reflect in vivo circumstances.^44^, ^45^, ^46^, ^47^ Variation in the viscoelastic characteristics of ex vivo tissues, as compared with in vivo tissues, may arise due to variations in cerebrospinal fluid pressure and tissue degradation. These disparities can occur even when the tissues are stored under ideal conditions for preservation.^48^, ^49^ Nevertheless, this should not impact the assessment of the relative behaviors of damaged and undamaged spinal tissues. In addition, this study did not investigate any particular mechanism of durotomy. Further investigation is necessary to examine the underlying mechanisms that elucidate the impact of early decompressive physiology on apoptosis, blood flow, and inflammatory changes in the chronic entry phase.

In summary, the findings suggested that prompt intrathecal decompression had a significant positive impact on the mobility of rats with thoracic SCI. Moreover, performing surgical durotomy following crush SCI led to a significant and enduring hindrance in the infiltration of activated macrophages inside and around the injury site, which was linked to an almost complete reduction of posttraumatic cystic cavitation. In conclusion, our findings demonstrate that durotomy significantly restricts the formation of fibrous scar tissue, as evidenced by a reduction in fibronectin deposition, and enhances the swift restoration of tissue elasticity. Performing an early surgical decompressive durotomy could potentially reduce secondary damage and establish a more favorable microenvironment for the potential transplantation of cells or biomaterials in the treatment of spinal cord injuries.

## 4 MATERIALS AND METHODS

### 4.1 Study design

All experimental techniques were carried out in accordance with the United Kingdom Animals (Scientific Procedures) Act of 1986. The animal experimentation techniques were authorized by the Animal Welfare Committee at Tongji Hospital, which is affiliated with Tongji University in Shanghai, China. Figure 1 depicts the study's design. This study involved the selection of 142 mature female Sprague–Dawley rats, aged 11−12 weeks and weighing between 240 and 260 g. The rats were allocated into three groups through a random process: the sham control group (*N* = 16), which underwent only laminectomy; the SCI + laminectomy group (epidural decompression, *N* = 63), which experienced clip‐compression injury along with laminectomy alone; and the SCI + durotomy group (intrathecal decompression, *N* = 63), which underwent clip‐compression injury along with laminectomy and decompressive durotomy. Rats were evaluated by behavioral testing, bladder function analysis, tissue water content analysis, histology analysis, MRI measurements, and mechanical indentation testing at different time points postinjury.

### 4.2 Surgical procedure of rat SCI model

Aseptic protocols were enforced for each surgical procedure. Anesthesia was initiated with the use of isoflurane (4.0% oxygen) and sustained at a concentration of 1.5–2.0% in oxygen delivered via a facemask. A surgical cut was created along the middle line of the body to access the thoracic vertebrae, and then a procedure called T8‐T10 laminectomy was performed. In order to avoid harm to the adjacent nerve roots, an aneurysm clip (50.0 g; Fine Science Tools, Heidelberg, Germany) was passed extradurally anterior to the cord, while still in its open position. Due to the rapid discharge of the clip from the applicator, an acute dorsoventral impact‐compression injury was established. The spinal cord was fully crushed at the T9 spinal‐cord level for 30 s (Figure 1A). Separate sham control animals were subjected to T8–T10 laminectomy; nevertheless, no sustained compressive injury was induced. Perform manual bladder expression twice daily until normal bladder function is restored. In this paper, the term “postinjury” will be employed to measure the amount of time that has passed for the procedures outlined above, including those performed on animals in the sham control group.

### 4.3 Surgical procedure of durotomy

After 24 h of injury, the sutures were removed in order to reveal the location of the compressed injury. Rats in both the control and SCI + laminectomy groups underwent the same procedure of having their incisions restitched and closed, following which they were given postoperative bladder expression, as previously mentioned. Rats that were randomized and assigned to undergo a decompressive durotomy had their surgical sites exposed again. The dura was incised using a #11 blade under high magnification, creating a durotomy with a diameter of around 5.0 mm. The dural opening was then gently expanded using two Dumond #5 forceps. Careful evacuation was performed to examine the exposed intradural spinal cord and remove any remaining bleeding or blood clots. In order to decrease excessive adhesion, a small adipose tissue fragment was placed on the top of the spinal cord. The incisions were closed, and care was provided as described above.

### 4.4 Behavioural analysis

The BBB open‐field locomotor rating scale is a highly responsive assessment of hindlimb locomotor behavior, which is evaluated by an observer.^50^ Ten rats randomly selected from each group were evaluated for blinded rating by two well‐trained observers on days 3, 7, 14, 21, and 28 postinjury.

### 4.5 Bladder function analysis

After thoracic SCI, manual bladder expression was necessary for all animals. For the purpose of maintaining records, each bladder expression was categorized based on its size, with observations varying from being empty to being of considerable size. Ten rats, randomly selected from each group, were evaluated for bladder function recovery. Urine from the first bladder expression of the day was collected and measured with a serological pipette on days 3, 7, and 14 postinjury. The average residual urine volumes were then calculated over a period of time to determine the recovery time of relative bladder function for each group in the experiment.

### 4.6 Water content analysis

After the surgery, a combined number of 48 creatures (SCI + laminectomy group [*N* = 24]; SCI + durotomy group [*N* = 24]) were put to sleep on the 3rd, 7th, and 14th days. A ∼20.0 mm long sample was obtained by cutting their spinal cords 10.0 mm toward the head and 10.0 mm toward the tail from the lesion site, after which it was isolated and removed. The cord segments were placed into Eppendorf tubes that had been preweighed and then weighed on an analytical scale to determine their wet weight. The sections of cord were placed in an oven at a temperature of 105.0°C for a duration of 24 h. Afterward, they were reweighed (as dry weight) to ascertain the amount of water that had evaporated. The water content percentage was determined by applying the Elliot equation:

(1) $$\mathrm{Percent}\;\mathrm{water}\;\mathrm{content}\;=\frac{\mathrm{wet}\;\mathrm{weight}-\mathrm{dry}\;\mathrm{weight}}{\mathrm{wet}\;\mathrm{weight}}\;\times 100\;$$

### 4.7 MRI experiment

All MRI measurements were performed on a dedicated small bore animal 11.7‐T scanner (Biospec 117/16; Bruker, Ettlingen, Germany) (Figure 1C). The detailed MRI parameters were presented in the Supplementary Material (Method S1).

### 4.8 MRI analysis and statistics

Figure 1D displays the measurement of lesion indicators on a sagittal T2‐weighted image. The lesion area refers to the proportion of the lesion region in the T8–T10 spinal cord, whilst the lesion length and breadth reflect the largest longitudinal and transverse diameters of the lesion area, respectively. BASIC scoring was conducted based on the observations made on an axial T2‐weighted picture as depicted in Figure 1E.^51^, ^52^

### 4.9 Tissue processing, hematoxylin–eosin staining, and immunohistochemistry

Coronal histological slices of the cord segments were prepared at a thickness of 4.0 mm and stained with hematoxylin and eosin (H&E) to examine the tissue's histological structure after the injury.

To evaluate the presence of inflammatory infiltration and the formation of scars, immunohistochemistry was conducted. Following three washes in PBS, the sections were then blocked with normal goat serum for a duration of 30 min and subsequently washed again using PBS. The sections were then left to incubate overnight with different primary antibodies: (1) rabbit anti‐CD68 (Servicebio; GB113109, 1:1000) for systemic macrophages, (2) rabbit anti‐CD11b (Servicebio; GB11058, 1:500) for macrophages/activated microglia, (3) rabbit anti‐GFAP (Servicebio; GB11096, 1:1000) for astrocytes, and (4) rabbit antifibronectin (Servicebio; GB13091, 1:100) for potent inhibitors of CNS regeneration. The primary antibodies were rinsed in PBS (3 × 5 min). Subsequently, the sections were exposed to goat anti‐rabbit horseradish peroxidase (Servicebio; GB23303, 1:500), goat anti‐rabbit Cy3 (Servicebio; GB21303, 1:300), and goat anti‐rabbit Alexa Fluor 488 (Servicebio; GB25303, 1:400) secondary antibodies for a duration of 50 min. The slides were then treated with a solution of 4′,6‐diamidino‐2‐phenylindole (DAPI) at ambient temperature (∼24.0°C) for a duration of 10 min while being stored in a light‐free environment. The sections were then rinsed with PBS (3 × 5 min), affixed using antifade mounting media, and secured with a coverslip. Fluorescence microscopy was employed to photograph sagittal slices of the spinal cord.

### 4.10 Morphological quantification

The quantification of CD68 stained regions was performed to evaluate the presence of inflammatory cells (Method S4). Immunofluorescence labelling with GFAP and fibronectin was used to assess the extent of scar formation at the site of injury (Method S4). Cavity areas were analyzed using serial sections stained with H&E (Method S4).

### 4.11 Indentation setup

Figure 1F depicts a diagram illustrating the indentation experiments. The mechanical elastic characteristics of the tissue were quantified using a Mach‐1 Model V500css device (Biomomentum Inc., Laval, QC, Canada). The detailed device parameters were presented in the Supplementary Material (Method S2).

### 4.12 Tissue preparation and indentation measurements

The spinal cord tissue was dissected and prepared using standard procedures before indentation testing, as previously described (Method S3).^53^

Indentation measurements were utilized to generate stiffness maps for the purpose of analyzing the spatial organization of the elastic modulus of the spinal cord. A total of 54 rats were used in the studies, with 24 rats in the SCI + laminectomy group, 24 rats in the SCI + durotomy group, and six rats in the control group. The indenter was given a spherical tip with a diameter of 0.50 mm. Animals with spinal crush injuries displayed inconsistent and dispersed borders, indicating different degrees of tissue injury. The Mach‐1 acquisition program was deployed to capture the force–displacement curves for every region of interest encompassed by a grid of indentation points. The indentation rate was 0.10 mm/s, and the measured depth was 0.25 mm. Sequential indentations were initiated at the front edge of the affected area (∼10.0 mm) and progressed toward the tail, with a spacing of 0.5 mm between each indentation, until the undamaged portions of the specimen, confirmed by visual examination, were reached. Each specimen had an average of around 39 recorded measures (Figure S3). Significantly, in the assessment of a nonlinear specimen response using a spherical indenter tip, it is of utmost importance to consistently and accurately determine the interaction between the indenter tip and soft tissue, as it has been observed that visually estimating the contact conditions is not dependable or precise.^54^ Therefore, a more organized method was created to avoid incorrect identification and consider minor changes in force (Figure S4).

### 4.13 Indentation data analysis

The determination of the elastic modulus was accomplished using the indentation technique and a spherical indenter, as illustrated in Figure 1G. As mentioned before, the Mach‐1 analysis software was employed to adapt the elastic model (Hayes's model) for indentation to the force–displacement curve.^55^ The elastic moduli at each place were determined, employing a Poisson's ratio of 0.5. The mean thickness of 3.0 mm (with a range of 2.8–3.2 mm) was determined using a digital caliper.^56^, ^57^, ^58^, ^59^ The mechanical characterization of the samples affixed to a level inflexible backing was highly appropriate for this framework (at minimum 10 times more rigid than the sample). The formula below was used to calculate the elastic modulus:

(2) $$\mathrm{Elastic}\;\mathrm{modulus}\;=\frac{P}{H}\;\times \frac{1-v^{2}}{2ak\left(\frac{a}{h},v\right)}\;$$

The variables in the equation are as follows: P represents the force applied, H represents the displacement caused by indentation, v represents Poisson's ratio, a represents the radius of the contact region, k determined by the a /h and v, and h represents the thickness of the specimen.

### 4.14 Statistical analysis

The data analysis was conducted using both SPSS and GraphPad Prism software. Utilizing a two‐tailed Student's *t*‐test to assess the disparities between the two groups and employing the Shapiro–Wilk normality assessment test to determine the normality of the data correspondingly. The Mann–Whitney–Wilcoxon test was utilized to compare results that deviated from a normal distribution. When the variances were equal, multiple group comparisons were performed using a one‐way analysis of variance (ANOVA) together with Bonferroni's post hoc analysis. The Kruskal–Wallis ANOVA with Games‐post *hoc* analysis was used in all other scenarios. ^*^ *p* < 0.05, ^**^ *p* < 0.01, and ^***^ *p* < 0.001.

## AUTHOR CONTRIBUTIONS

C. J., L. C., and N. X. designed the study. C. J. and Y. R. performed the experiments. C. J., Y. R., Z. W., Y. L., and K. W. analyzed the data. C. J. drafted the manuscript. C. J., Y. R., L. C., and N. X. revised the paper. All authors read and approved the final manuscript.

## CONFLICT OF INTEREST STATEMENT

The authors declare that they have no competing interests.

## ETHICS STATEMENT

All experimental animal ethics are approved by the Animal Welfare Committee of Tongji Hospital, affiliated with Tongji University in Shanghai, China (No. 2021‐DW‐012).

## Supporting information

Supporting Information

## Data Availability

All data generated or analyzed during this study are included in this published article and its Supplementary Materials.
