# Supplementary material for: Role of durotomy on function outcome, tissue sparing, inflammation, and tissue stiffness after spinal cord injury in rats
Source: MedComm (2020). 2024 Apr 4;5(4):e530. doi: 10.1002/mco2.530 (PMC10993870; doi:10.1002/mco2.530)
Supplement: Supplementary file 1 — Supporting Information [file MCO2-5-e530-s001.docx]

Supplementary Materials for

**Role of durotomy on function outcome, tissue sparing, inflammation and tissue stiffness after spinal cord injury in rats**

Chen Jin, Kaiwei Wang, Yilong Ren, Yi Li, Zhanwei Wang, Liming Cheng^*^, Ning Xie^*^

*Corresponding authors. E-mail: limingcheng@tongji.edu.cn (L.C.); nxieprof18@tongji.edu.cn (N.X.)

**This file includes:**

Methods S1 to S4

Figures S1 to S4

Tables S1 to S5

**Method S1** **The detailed MRI parameters**

The MRI scanner was operated with a maximum gradient strength of > 740 mT/m and a slew rate of 6660 T/m/s in all three directions. Sagittal T2-weighted fast spin echo imaging was performed with the following parameters: TE/TR 20/600 ms (TE = echo time, TR = repetition time), slice thickness 1.0 mm, spacing 1.2 mm, acquisition matrix 240 × 120, echo train length 6, and NEX 15. Imaging using the T2-weighted fast spin echo technique was conducted in the axial plane with the specified settings: TE/TR 20/630 ms, slice thickness 1.2 mm, spacing 1.5 mm, acquisition matrix 200 × 200, echo train length 6, and NEX 10. The phase-encoding direction for axial sequences was from left to right, whereas it was from craniocaudal for sagittal sequences.

**Method S2** **The detailed device parameters of Mach-1 Model V500css device**

Employing a solitary axial load cell to gauge the perpendicular force and precisely evaluate the surface inclination at each site. The measurement range on the vertical axis is 1.50 N with a resolution of 0.07 mN. The load cell's performance parameters, namely linearity, hysteresis, as well as non-repeatability, were ±0.15% full scale, ±0.15% full scale, and ±0.10% full scale, respectively. The force signal that was measured underwent conversion into a digital value and was subsequently transferred to a computer via a load cell amplifier, which provided power to the load cell.

**Method S3** **Tissue preparation**

Briefly, rats were euthanized and perfused at 3, 7, 14, and 28 days post injury. Following that, a surgical incision was performed at the junction between the seventh cervical and first lumbar vertebrae. The extracted thoracic spinal cord segments were promptly placed in PBS. Afterward, a culture dish with a diameter of 100.0 mm was filled with cord tissue obtained from ex vivo, which covered the injury site. The tissue was separated from the surrounding dura with the help of thin forceps. It was then placed on a slide with the ventral surface facing upwards and attached employing a cyanoacrylate adhesive. In order to avoid tissue deterioration, samples were moisturized with cold PBS. Before indentation, a 10-minute acclimation period was permitted. Afterward, the petri dish was positioned below the indenter tip. Indentation tests were performed within one hour of euthanasia at room temperature (~24.0 °C) due to the significant impact of postmortem time on the mechanical properties of biological tissues.

**Method S4 Morphological quantification**

The quantification of CD68 stained regions was performed to evaluate the presence of inflammatory cells utilizing the ImageJ software. Five sections from each rat, totaling 10% of the longitudinal sections, were processed for each group. Cells that were labeled with immunolabeling were transformed into a region of interest (ROI) under a 200× magnification.

Immunofluorescence labelling with GFAP and fibronectin was used to assess the extent of scar formation at the site of injury. For the research, a single longitudinal section was chosen from every 10 animals. Following the application of immunostaining using the corresponding indicators, five randomly selected areas (magnified at 200×) were designated for imaging and analysis.

Cavity areas were analyzed using serial sections stained with H&E. H&E staining was performed on chosen sections and captured in photographs. Cavities located within a 3 mm radius of the injury site in either the front or back area were assessed, and any cavities smaller than 160 μm in diameter were not considered. For each group, the average of the total area of cavities in every slice was calculated for each experimental animal.


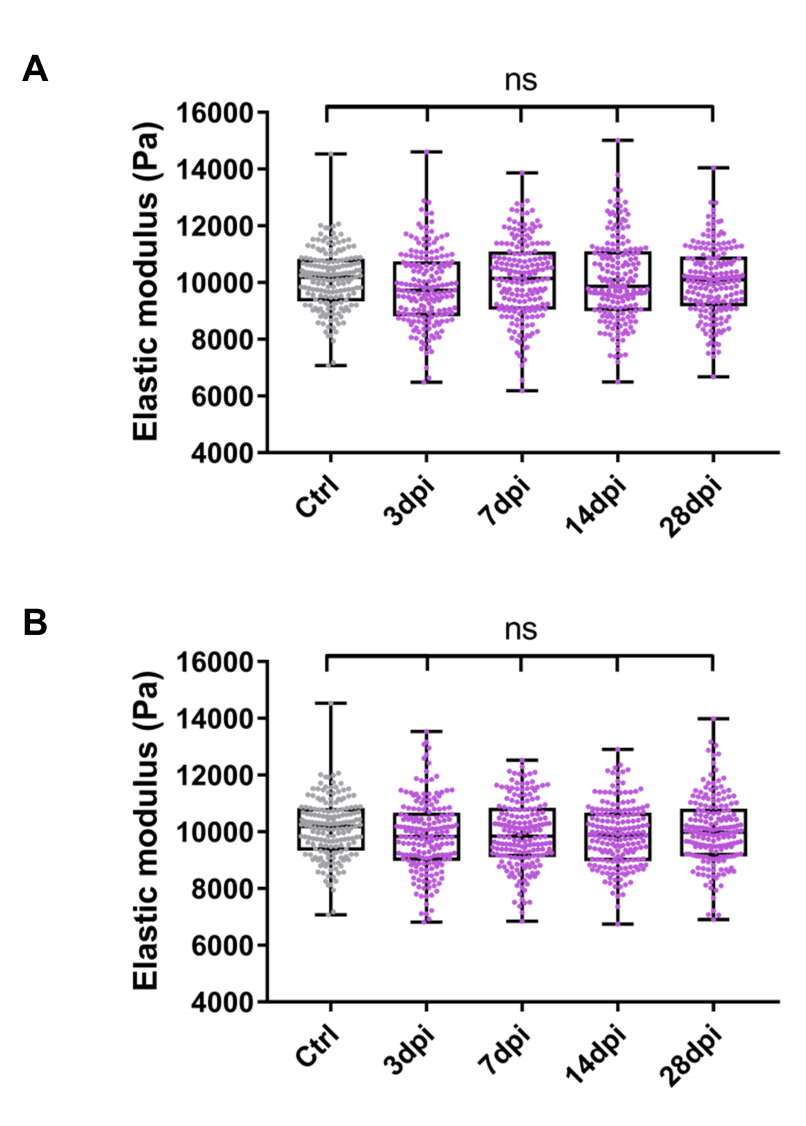


Figure S1 Comparison of the elastic moduli between uninjured spinal cord tissue and the sham controlled group at different time points postinjury in SCI + laminectomy group (A) and SCI + durotomy group (B). The elastic moduli of spinal cord tissue from sham controlled rat displays relatively constant stiffness values and were used as a reference for uninjured spinal cord tissue. In all box plots, the top and bottom of the box represent the 75th and 25th percentiles respectively and the line inside corresponds to the median. The filled small black cross denotes mean. Ctrl, control group; Dpi, days postinjury.


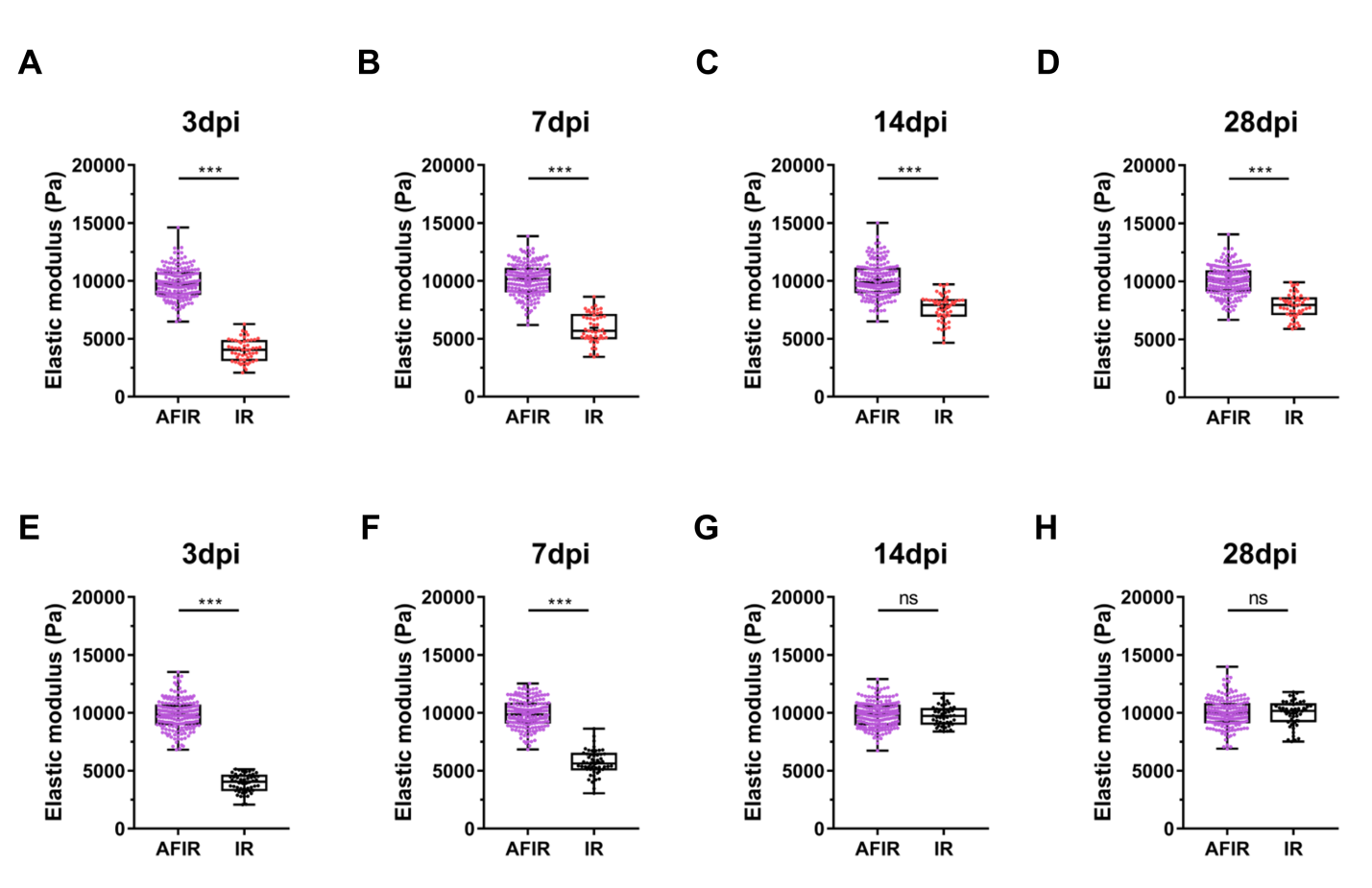


Figure S2 Comparison of the elastic moduli of the injury region and away from injury region at different time points after crush injury in SCI + laminectomy group (A-D) and SCI + durotomy group (E-H). In all box plots, the top and bottom of the box represent the 75th and 25th percentiles respectively and the line inside corresponds to the median. The filled small black cross denotes mean. Ctrl, control group; AFIR, away from injury region; IR, injury region; Dpi, days postinjury. ^***^ indicates *P* < 0.001.


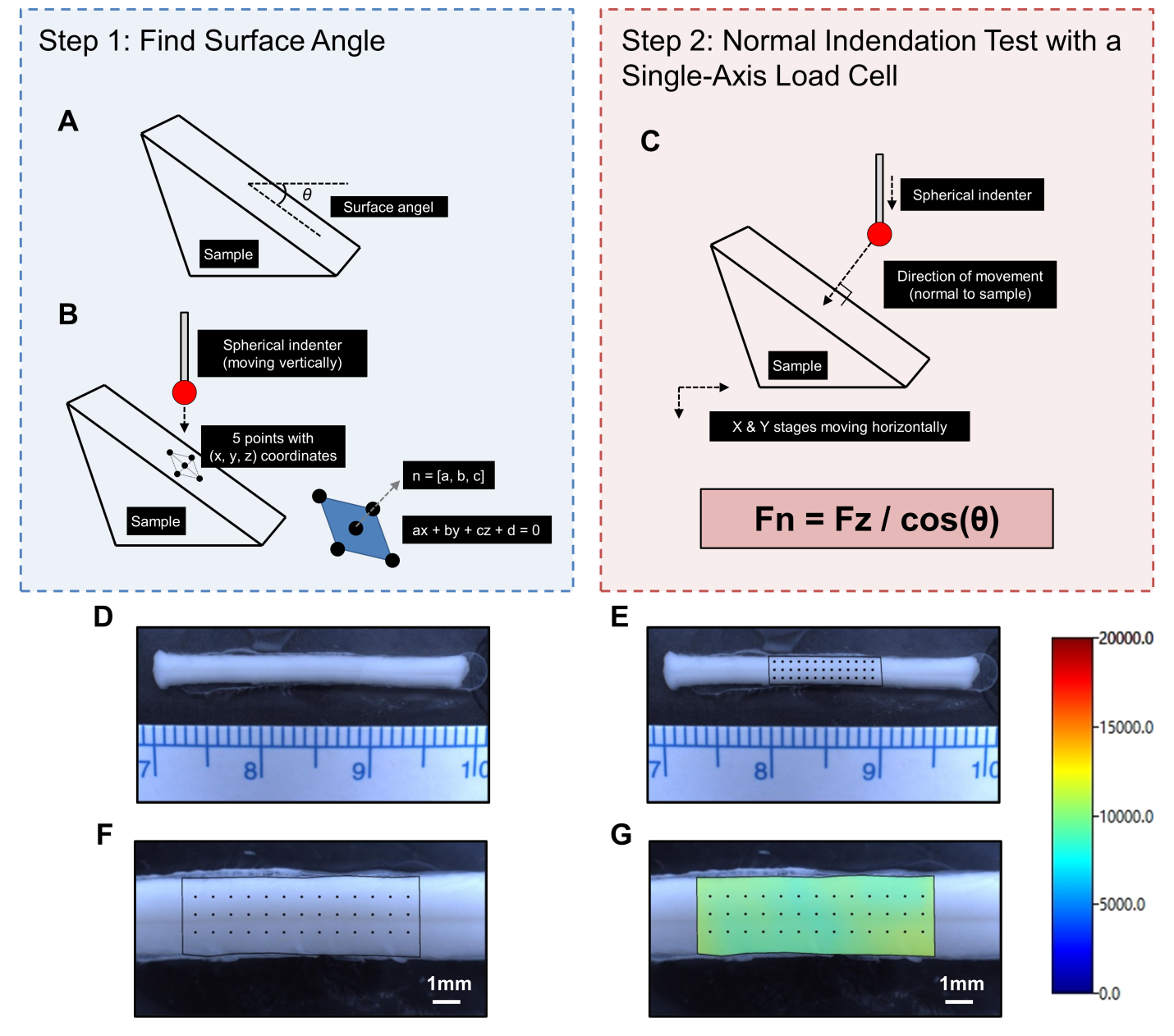


Figure S3 Normal indentation visual description. (A) Probe multiple points on sample. (B) Fit plane to points by least-squares method to find normal vector. (C) Move indenter along normal vector using all three stages. Using a single-axis load cell for normal indentation, the normal force is calculated using Fz and the previously found surface angle. (D-G) An example to identify the coordinates of each testing location for automated indentation mapping.


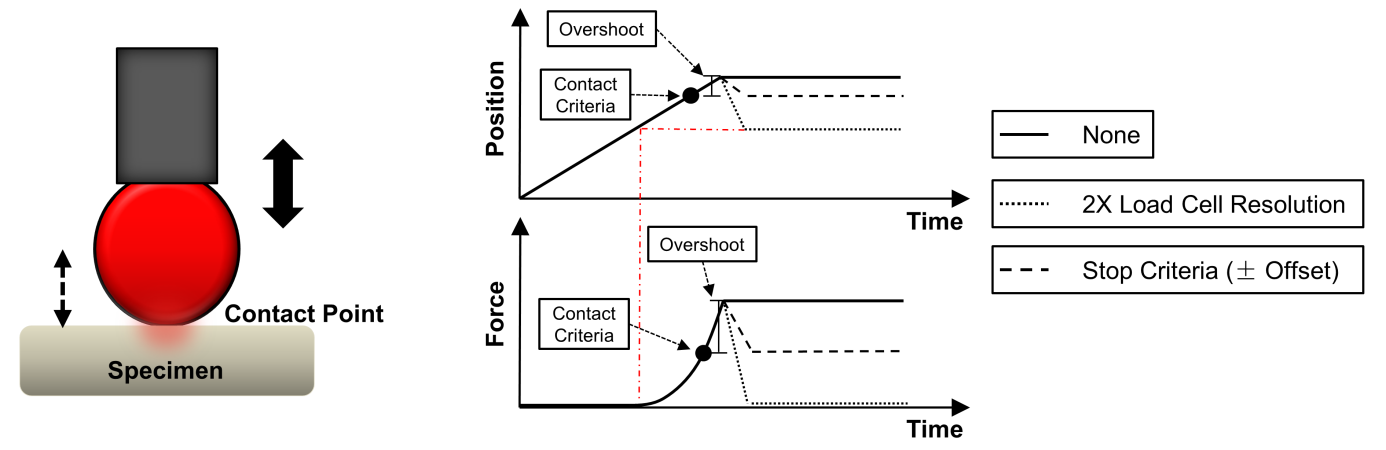


Figure S4 Tissue contact protocol. Schematic of indenter tip position relative to specimen surface during contact determination procedure ex vivo (the left diagram). Depending on the predefined stage velocity, the stage will continue to move (position will overshoot), more or less, following the reaching of the stop criteria (hardware delay) (the right diagram). In order to compensate for this overshoot, this function automatically analyses the force-displacement curve generated and repositions the stage based on the selected stage repositioning option. “None”, the stage remains at the position where it has stopped (overshoot). “Stop Criteria”, the stage is repositioned where the load variation first corresponded to the stop criteria (overshoot compensation). “2X Load Resolution”, the stage is repositioned where the load variation first corresponded to twice the load cell resolution (surface detection).

Table S1 Statistical comparison of lesion length (%), lesion width (%) and lesion area (%) between SCI + laminectomy and SCI + durotomy group on days 7 and 28 postinjury.

|  | **Lesion length (%)** | | **Lesion width (%)** | | **Lesion area (%)** | |
| --- | --- | --- | --- | --- | --- | --- |
|  | **7 days** | **28 days** | **7 days** | **28 days** | **7 days** | **28 days** |
| **SCI** | 35.3 ± 3.5 | 26.2 ± 3.1 | 76.3 ± 3.0 | 61.8 ± 2.8 | 39.7 ± 5.2 | 21.8 ± 4.5 |
| **Durotomy** | 24.9 ± 4.42 | 13.7 ± 2.9 | 53.9 ± 12.1 | 37.9 ± 8.9 | 17.4 ± 6.4 | 8.9 ± 3.2 |
| ***P* value** | 0.033^†^ | 0.007^†^ | 0.036^†^ | 0.011^†^ | 0.009^†^ | 0.015^†^ |

Values are presented as the mean ± standard deviation. † According to independent samples *t*-test. This table corresponds to Figure 3B-D.

Table S2 Statistical comparison of percent water content (%) between SCI + laminectomy and SCI + durotomy group.

|  | **Baseline** | **3 days** | **7 days** | **14 days** |
| --- | --- | --- | --- | --- |
| **SCI** | 64.1 ± 0.7 | 76.4 ± 3.5 | 72.2 ± 2.4 | 67.1 ± 1.7 |
| **Durotomy** | 64.5 ± 0.8 | 71.0 ± 2.5 | 68.5 ± 1.3 | 65.7 ± 1.0 |
| ***P* value** | 0.380^†^ | 0.011^†^ | 0.007^†^ | 0.111^†^ |

Values are presented as the mean ± standard deviation. † According to independent samples *t*-test. This table corresponds to Figure 3F.

Table S3 Statistical comparison between the elastic moduli of the injury region and away from injury region at different time points after crush injury in SCI + laminectomy group.

| Elastic modulus (Pa) | 3 days | 7 days | 14 days | 28 days |
| --- | --- | --- | --- | --- |
| AFIR | 9822.9 ± 1294.6 | 10120.4 ± 1370.6 | 10099.2 ± 1449.7 | 10046.8 ± 1218.6 |
| IR | 3986.9 ± 938.9 | 5951.5 ± 1253.6 | 7657.4 ± 1141.2 | 7889.8 ± 1039.2 |
| *P* value | < 0.001^†^ | < 0.001^†^ | < 0.001^†^ | < 0.001^†^ |

Values are presented as the mean ± standard deviation. † According to independent samples *t*-test. AFIR, away from injury region. IR, injury region. This table corresponds to Figure S2A-D.

Table S4 Statistical comparison between the elastic moduli of the injury region and away from injury region at different time points after crush injury in SCI + durotomy group.

| Elastic modulus (Pa) | 3 days | 7 days | 14 days | 28 days |
| --- | --- | --- | --- | --- |
| AFIR | 9850.5 ± 1270.9 | 9923.3 ± 1170.4 | 9859.6 ± 1089.5 | 10018.5 ± 1191.9 |
| IR | 3943.4 ± 775.9 | 5706.2 ± 1081.3 | 9733.5 ± 816.2 | 9955.0 ± 1087.2 |
| *P* value | < 0.001^†^ | < 0.001^†^ | 0.467^†^ | 0.734^†^ |

Values are presented as the mean ± standard deviation. † According to independent samples *t*-test. AFIR, away from injury region. IR, injury region. This table corresponds to Figure S2E-H.

Table S5 Statistical comparison of elastic moduli (Pa) of the injury region between SCI + laminectomy and SCI + durotomy group on days 3, 7, 14 and 28 postinjury.

|  | **3 days** | **7 days** | **14 days** | **28 days** |
| --- | --- | --- | --- | --- |
| **SCI** | 3986.9 ± 938.9 | 5951.5 ± 1253.6 | 7657.4 ± 1141.3 | 7889.8 ± 1039.2 |
| **Durotomy** | 3943.4 ± 775.9 | 5706.2 ± 1081.3 | 9733.5 ± 816.2 | 9955.0 ± 1087.2 |
| ***P* value** | 0.796^†^ | 0.293^†^ | < 0.001^†^ | < 0.001^†^ |

Values are presented as the mean ± standard deviation. † According to independent samples *t*-test. This table corresponds to Figure 7E.
